# Liraglutide treatment attenuates inflammation markers in the cardiac, cerebral and renal microvasculature in streptozotocin‐induced diabetic rats

**DOI:** 10.1111/eci.13807

**Published:** 2022-05-07

**Authors:** Umit Baylan, Amber Korn, Reindert W. Emmens, Casper G. Schalkwijk, Hans W. M. Niessen, Paul A. J. Krijnen, Suat Simsek

**Affiliations:** ^1^ Department of Pathology Amsterdam UMC location VUmc Amsterdam the Netherlands; ^2^ Amsterdam Cardiovascular Sciences Amsterdam the Netherlands; ^3^ Department of Internal Medicine Maastricht University Medical Centre Maastricht the Netherlands; ^4^ Cardiovascular Research Institute Maastricht (CARIM) Maastricht the Netherlands; ^5^ Department of Internal Medicine Alkmaar the Netherlands; ^6^ Department of Internal Medicine Amsterdam UMC location VUmc Amsterdam the Netherlands

**Keywords:** advanced glycation endproducts, cerebral vasculature, CML, diabetes mellitus, GLP‐1 analogue, ICAM‐1 (CD54), intramyocardial vasculature, Liraglutide, NADPH oxidases, renal vasculature, VCAM‐1 (CD106)

## Abstract

**Background:**

Diabetes mellitus (DM) induces cardiac and cerebral microvascular dysfunction via increased glycation, oxidative stress and endothelial activation. Liraglutide, a glucagon‐like peptide‐1 analogue, inhibited NOX2 and adhesion molecules in isolated endothelial cells. Here, we have studied how Liraglutide affects advanced glycation, NOX expression and inflammation of the cardiac, cerebral and renal microvasculature in diabetic rats.

**Methods:**

DM was induced in Sprague–Dawley rats (*n* = 15) via intraperitoneal streptozotocin (STZ) injection (60 mg/kg bodyweight). Ten control rats remained nondiabetic. From day 9 post‐STZ injection, Liraglutide (200 μg/kg bodyweight; *n* = 7) or vehicle (*n* = 8) was injected subcutaneously daily until termination on day 29. The advanced glycation endproduct N‐ε‐(carboxymethyl)lysine (CML), NOX2, NOX4, ICAM‐1 and VCAM‐1 were subsequently immunohistochemically analysed and quantified to compare Liraglutide treatment with placebo.

**Results:**

In the heart, Liraglutide treatment significantly reduced the DM‐increased scores/cm^2^ for CML in both ventricles (from 253 ± 53 to 72 ± 12; *p* = .003) and atria (343 ± 29 to 122 ± 8; *p* = .0001) and for NOX2, ICAM‐1 and VCAM‐1, but not for NOX4. Also in the cerebrum and cerebellum of the brain, Liraglutide significantly reduced the scores/cm^2^ for CML (to 60 ± 7 (*p* = .0005) and 47 ± 13 (*p* = .02), respectively), and for NOX2 and NOX4. In the kidney, the DM‐induced expression of ICAM‐1 and VCAM‐1 was decreased in the blood vessels and glomeruli by Liraglutide treatment. Liraglutide did not affect blood glucose levels or bodyweight.

**Conclusions:**

Our study implies that Liraglutide protects the cardiac, cerebral and renal microvasculature against diabetes‐induced dysfunction, independent of lowering blood glucose in a type 1 diabetes rat model.

## 1 INTRODUCTION

Diabetes mellitus (DM) is associated with an increased risk of cardiovascular disease and can among others affect the heart and the brain, leading to and/or aggravating diseases including heart failure, atrial fibrillation and diabetic encephalopathy.^1^, ^2^, ^3^ Microvascular dysfunction is thought to be a major contributor herein, as indicated by a decrease in coronary flow velocity reserve, impaired microvascular vasodilator function and increased microcirculatory resistance in the heart and brain of DM patients.^2^, ^4^, ^5^ It is suggested that microvascular dysfunction progresses from functional impairment in early‐stage DM to structural impairment thereafter.^6^ Early therapeutic targeting of microvascular dysfunction in DM might therefore prevent the development of heart and brain disease.

The incretin glucagon‐like peptide 1 (GLP‐1) might be a suitable therapeutic candidate to counteract DM‐induced microvascular dysfunction.^7^ Synthetic analogues of GLP‐1, such as Liraglutide and Exenatide, have established glucose‐lowering effects and can exert cardiovascular protective effects both related to DM and non‐DM. The LEADER trial showed that Liraglutide‐treated type 2 diabetes patients had a lower rate of death from cardiovascular causes, nonfatal myocardial infarction or nonfatal stroke and a lower rate of cardiovascular death and renal outcomes compared with placebo.^8^ In addition, GLP‐1 analogues have been shown to improve endothelial and (micro)vascular function both in vitro^9^ and in vivo in atherosclerotic mice.^10^, ^11^ However, whether and how Liraglutide treatment protects the cardiac, cerebral and renal microvasculature from DM‐induced dysfunction‐associated changes in vivo is not known.

Elevated microvascular advanced glycation endproducts (AGEs) have been implicated as important drivers of DM‐induced microvascular dysfunction in the heart,^12^ as they can increase (micro)vascular stiffness and activate intracellular signalling pathways that lead to increased oxidative stress, permeability and inflammation.^13^ AGEs can be formed through the naturally occurring nonenzymatic glycation of proteins, lipids and nucleic acids. Previously, we observed increased accumulation of the AGE N‐**ε**‐(carboxymethyl)lysine (CML) in the cardiac and cerebral microvasculature of DM patients and diabetic rats.^14^ Moreover, cardiac CML accumulation correlated with left ventricular dysfunction in diabetic patients,^15^ while CML accumulation in brain vessels related to cognitive impairment^16^.

Advanced glycation endproducts accumulation is also associated with oxidative stress and inflammation. The reactive oxygen species (ROS)‐producing NADPH oxidase (NOX) proteins are important drivers of DM‐induced oxidative stress^17^ and have been shown to affect AGE accumulation in turn^18^, and increase the expression of the proinflammatory intercellular adhesion molecule 1 (ICAM‐1) and vascular cell adhesion molecule 1 (VCAM‐1)^19^, ^20^, ^21^. Both ICAM‐1 and VCAM‐1 showed increased gene expression in the kidney of diabetic rats^22^. These studies thus point to important roles for AGEs, NOX proteins and inflammation in DM‐induced cardiac, cerebral and renal microvascular dysfunction.

In the present study, we therefore studied the effect of Liraglutide therapy on microvascular AGE formation, NOX protein expression and ICAM‐1/VCAM‐1 expression in the hearts, brains and kidneys of diabetic rats.

## 2 MATERIALS AND METHODS

### 2.1 Animal experiment

We used 25 male Sprague–Dawley rats (aged 8 weeks) in total, divided into 3 experimental groups: a nondiabetic group (control; *n* = 10), a diabetic group (DM; *n* = 8) and a diabetic group with Liraglutide treatment (DM + LG; *n* = 7). On day one of the experiment, DM was induced with a single intraperitoneal streptozotocin injection (STZ; Sigma; 60 mg/kg bodyweight^14^). On day 9 glucose levels were determined in venous blood from the tail vein using a glucose meter (FreeStyle Precision, Abbott). Rats (*n* = 15) with a blood glucose level above 13.9 mM were included in the experiment. Liraglutide (Novo Nordisk) treatment started on day 9 and was given as daily subcutaneous injections (200 μg/kg bodyweight^23^, ^24^). Nontreated DM rats received similar daily vehicle injections. The bodyweight of all rats was determined twice a week throughout the experiment. On day 29 blood was obtained from the left ventricle of the heart, which was used to determine blood glucose levels, after which the rats were terminated. The hearts and brains were excised and fixed in 4% formaldehyde. The ventricles and atria of the heart and the cerebrum and cerebellum of the brain were then separately embedded in paraffin. The animal experiments were performed in compliance with the European Community guidelines for the use of experimental animals and approved by the National Authorities for Animal Experiments. Reporting of the study conforms to broad EQUATOR guidelines.^25^

### 2.2 Immunohistochemistry

The paraffin‐embedded tissue was cut into 4 μm thick sections and mounted onto microscope slides. The slides were first deparaffinised in xylene for 10 min, dehydrated in 100% ethanol for 10 min and incubated in methanol containing 0.3% H_2_O_2_ for 30 min to block endogenous peroxidases. Antigen retrieval was performed by incubating the slides in 0.1% pepsin buffer for 30 min at 37°C (for CML staining) or boiling in citrate buffer (pH 6.0; for NOX4 staining) and TRIS/EDTA buffer (pH 9.0) for 10 min (for NOX2, ICAM‐1 and VCAM‐1 staining). All sera and antibodies were diluted in Normal Antibody Diluent Solution (NAD; ImmunoLogic) and incubations were at room temperature unless otherwise specified. The slides were blocked in either 2% w/v bovine serum albumin (BSA; for CML staining), normal swine serum (1:10 dilution; ImmunoLogic; for NOX4 staining), or normal rabbit serum (1:10 dilution; Dako) for 10 min. Subsequently, slides were incubated with mouse‐anti‐human CML antibody (1:500 dilution; Baidoshvili et al., 2006), mouse‐anti‐human NOX2 (1:10 dilution; Krijnen et al., 2003), rabbit‐anti‐rat NOX4 (1:100 dilution, Novus Biologicals), mouse‐anti‐rat ICAM‐1 antibody (1:50 dilution; Santa Cruz Biotechnology), or mouse‐anti‐rat VCAM‐1 antibody (1:100 dilution; Santa Cruz) for 1 h. Slides were rinsed with phosphate‐buffered saline (PBS) and incubated with a biotin‐conjugated swine‐anti‐rabbit antibody (1:300 dilution; Dako; for NOX4 staining) or rabbit‐anti‐mouse antibody (1:500 dilution; Dako) for 30 minutes, followed by another PBS rinse. Slides were subsequently either incubated with streptavidin‐HRP complex (1:500 dilution; Dako) for 1 hour for CML staining, with the ABC‐kit (1:100 dilution; Vector Lab) for 1 hour for NOX2 and NOX4 or with Envision HRP Rabbit (Dako) for 30 min for ICAM‐1 and VCAM‐1. Following visualisation with 3,3′ diaminobenzidine (DAB; Dako) for 8–10 min, slides were counterstained with haematoxylin, dehydrated in 100% ethanol and covered.

### 2.3 Tissue analysis

Quantification of the CML staining was performed with an intensity scoring method, whereby each CML‐positive blood vessel was given an intensity score of weak (1), moderate (2), or strong (3) positive.^26^ Each intensity score was multiplied by the number of blood vessels positive for this score and subsequently added, thus obtaining an immunohistochemical (IH) score. The IH score was then divided by the surface area of the analysed tissue resulting in an IH score per cm^2^, which can provide an overall view of the effects on CML. Additionally analysing the different intensities separately can provide further information on how CML is affected (i.e. change in the amount of CML‐positive vessels or change in CML accumulation in the same amount of vessels), thus we also analysed the number of blood vessels with a particular intensity score (weak (1), moderate (2), or strong (3)) per cm.^2^ The tissue surface area was determined using QuickPhoto 3.0 software (Promica, Prague, The Czech Republic) on scanned slides (PathScan Enabler IV slide scanner, Meyer Instruments). The number of NOX2, NOX4, ICAM‐1 and VCAM‐1 positive blood vessels were counted and divided by the tissue surface area, representing the number of positive blood vessels per cm.^2^ The kidney tissue was analysed by selecting 5 tiles of 2 mm^2^ on the scanned slides (Digipath (Philips, v3.3), in which the number of ICAM‐1 and VCAM‐1 positive blood vessels were counted. Subsequently, the total number of positive ICAM‐1 and VCAM‐1 blood vessels was divided by the total tissue surface area (10 mm^2^), thus representing the number of positive blood vessels per mm.^2^ In the same tiles the number of ICAM‐1 and VCAM‐1 positive glomeruli was counted and divided by the total number of glomeruli, thus resulting in a percentage of ICAM‐1 and VCAM‐1 positive glomeruli.

### 2.4 Statistical analysis

Data analysis was performed with Prism v.4.0 (Graphpad Software). Differences between groups were analysed with a one‐way ANOVA and Tukey's multiple comparisons test when data were normally distributed, and with a Kruskal‐Wallis test and Dunn's multiple comparisons test when data were not normally distributed. Data are represented as mean ± standard error. A *p*‐value < .05 was considered statistically significant for all performed analyses.

## 3 RESULTS

### 3.1 Bodyweight measurements

On day 1 there were no significant differences in bodyweight between the groups. The average bodyweight of control rats increased significantly from 286 grams on day 1–314 g on day 29 (*p* < .05). In contrast, the average bodyweight of the DM and DM + LG rats decreased from 281 and 292 g, respectively, on day 1–262 (*p* = .04) and 265 g (*p* = .003), respectively, on day 29. The average bodyweight on day 29, nor the weight loss compared with day 1, differed significantly between the DM and DM + LG rats, but both groups significantly differed from the control rats (DM: *p* = .0001; DM + LG: 0.0004; Table 1).

**TABLE 1:** Bodyweight and blood glucose of all rats included

	Bodyweight (g)		Blood glucose (mM)	
	Day 1	Day 29	Day 9	Day 29
Control	286 ± 5	314 ± 6^††††^	5.7 ± 0.1	13.1 ± 0.8^††††^
DM	281 ± 3	26s2 ± 9^†,^*	26.4 ± 1.1****	27.2 ± 0.2***
DM + LG	292 ± 3	265 ± 8^††,^*	22.7 ± 1.8*	25.0 ± 2.4*

Note

Bodyweight presented in grams and blood glucose levels presented in mM of control (*n* = 10), DM (*n* = 8) and DM + LG rats (*n* = 7). Experimental groups DM and DM + LG were compared with the control on the same day (displayed with *) using a Kruskal–Wallis test, and all experimental groups were compared between days (displayed with ^†^) using a paired *t*‐test. Data are displayed as mean ± SEM. *p* < .05*, *p* < .01**, *p* < .001***, *p* < .0001****.

### 3.2 Blood glucose measurements

In the control group, the blood glucose levels were 5.7 ± 0.1 mM on day 9, which increased significantly to 13.1 ± 0.8 mM (*p* < .0001) on day 29 (Table 1). The blood glucose levels of both the DM and the DM + LG rats on day 9 were significantly higher than the control rats (26.4 ± 1.1 mM, *p* < .0001; and 22.7 ± 1.8 mM, *p* = .01, respectively). On day 29, the blood glucose levels were at similar levels as on day 9 in both the DM and DM + LG groups and were significantly higher than the blood glucose levels of the non‐DM group (DM: *p* = .006; DM + LG: *p* = .01).

### 3.3 Liraglutide attenuates DM‐induced CML, NOX2, ICAM‐1 and VCAM‐1 accumulation in the cardiac microvasculature

**FIGURE 1:**
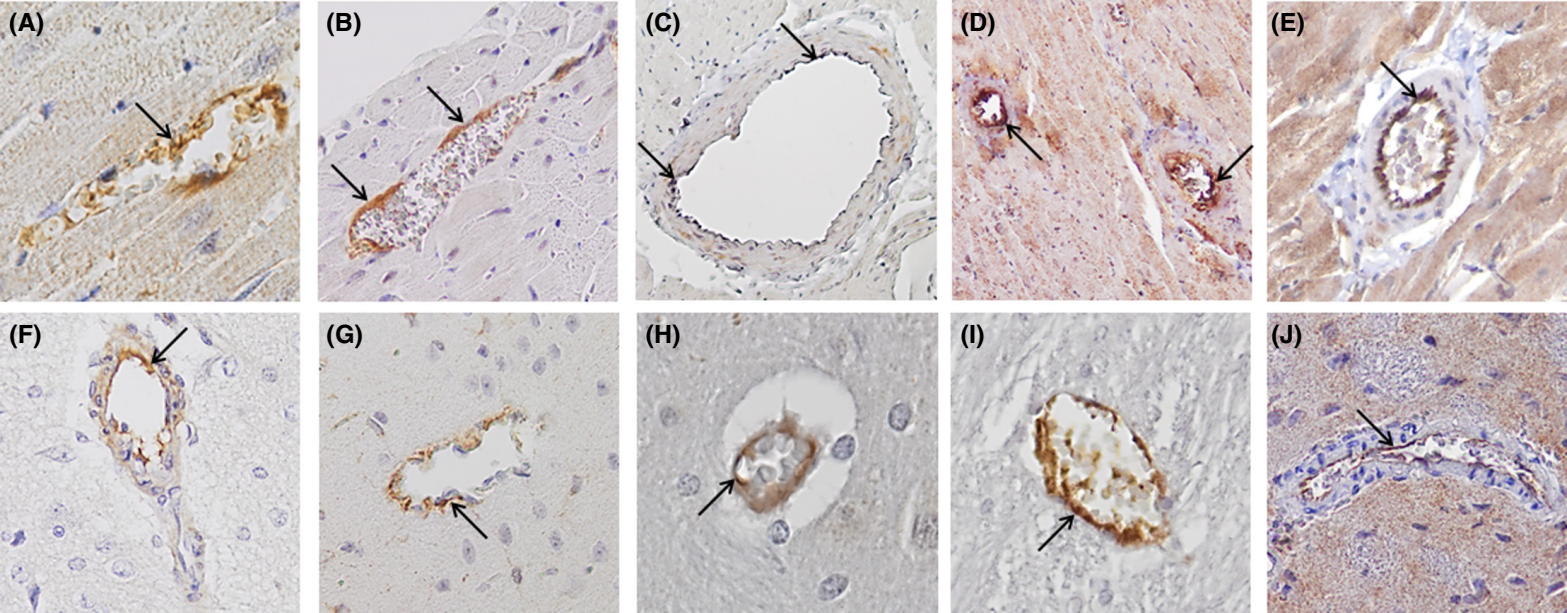
Immunohistochemical pictures of the inflammatory markers in intramyocardial and cerebral vasculature. Examples of CML (intensity score 2; A), NOX2 (B), NOX4 (C), ICAM‐1 (D) and VCAM‐1 (E) staining in intramyocardial blood vessels and cerebral blood vessels (F–J, respectively). Original magnification: (C and D) 100×, (B, E, F, G, I and J) 200×, (A) 400×. Arrows indicate staining of endothelial cells

In Figure 1 examples are shown of the immunohistochemical stainings of CML, NOX2, NOX4, ICAM‐1 and VCAM‐1 in the endothelium of intramyocardial blood vessels.

#### 3.3.1 CML

**FIGURE 2:**
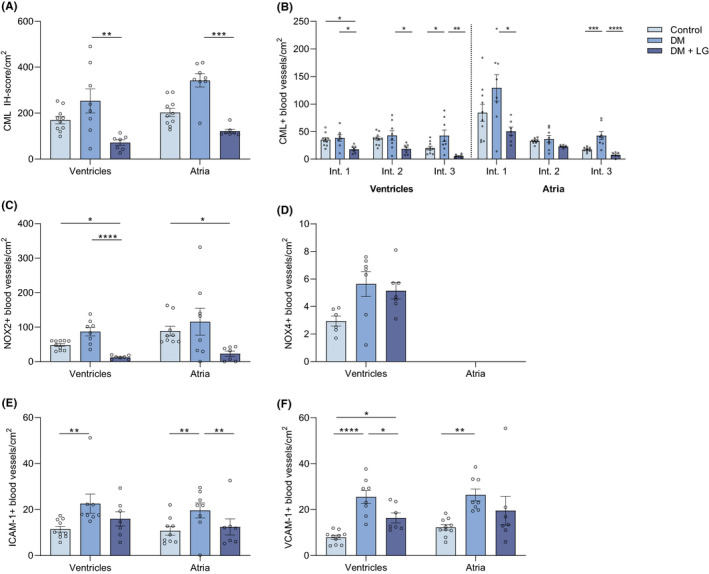
Liraglutide treatment decreases CML, NOX2, ICAM‐1 and VCAM‐1 accumulation in the cardiac microvasculature of diabetic rats. The immunohistochemical score/cm^2^ for CML ((A) defined in Methods) and the number of positive blood vessels/cm^2^ for NOX2 (C), NOX4 (D), ICAM‐1 (E) and VCAM‐1 (F) of the microvasculature of ventricles and atria in control rats, STZ‐induced diabetic rats (DM) and Liraglutide‐treated DM rats (DM + LG). The total number of blood vessels with a particular CML intensity score are separately depicted in (B): weak (score 1), moderate (score 2) and strong (score 3) intensity score. A one‐way ANOVA was used for analysis; data are represented as mean ± standard error. *p* < .05*, *p* < .01**, *p* < .001***, *p* < .0001****

Diabetes induced an increase in the average CML IH score/cm^2^ from 170 ± 16 in the ventricles and 203 ± 18 in the atria of control rats to 253 ± 53 in the ventricles and 343 ± 29 in the atria in the DM group (Figure 2A). Liraglutide treatment led to a significantly lower IH score/cm^2^ (ventricles: 72 ± 12, *p* = .003; atria: 122 ± 8, *p* = .0001) compared the nontreated DM rats, that was even below control levels. Analysis of the CML intensity scores revealed that, compared with controls, DM rats showed a significant increase especially in strong‐positive blood vessels (intensity score 3) both in the ventricles (*p* = .04) and atria (*p* = .0005) (Figure 2B). Liraglutide treatment significantly lowered the number of CML+ blood vessels among all intensity scores in the ventricles (score 1: *p* = .02, score 2: *p* = .02, score 3: *p* = .002) and those with intensity score 1 (*p* = .02) and score 3 (*p* < .0001) in the atria.

#### 3.3.2 NOX2 and NOX4

Diabetes also increased the average number of NOX2+ blood vessels/cm^2^ to 87 ± 12 in the ventricles and 116 ± 39 in the atria, compared with the control rats (ventricles: 48 ± 4; atria: 89 ± 14), albeit not significantly (Figure 2C). Liraglutide treatment decreased the number of NOX2+ blood vessels (ventricles: 13 ± 5; atria: 23 ± 8), which was significant compared with nontreated DM rats in the ventricles (*p* < .0001) and the controls in the ventricles (*p* = .02) and atria (*p* = .01). Also, the number of NOX4+ blood vessels/cm^2^ was increased in the ventricles of the DM group compared with the control group, albeit not significantly. In the atria, no NOX4+ blood vessels could be found. In contrast to NOX2, Liraglutide treatment did not affect NOX4 in the ventricles (Figure 2D).

#### 3.3.3 ICAM‐1 and VCAM‐1

Diabetes induced a significant increase in the number of ICAM‐1+ blood vessels/cm^2^ (controls: 12 ± 1 in the ventricles and 11 ± 2 in the atria; DM group: 23 ± 4 in the ventricles (*p* = .007) and 22 ± 2 in the atria (*p* = .02) (Figure 2E). Liraglutide treatment decreased number of ICAM‐1+ blood vessels/cm^2^ compared with the DM group, although only significantly in the atria (*p* = .04). Similarly, the number of VCAM‐1+ blood vessels/cm^2^ was significantly increased in the DM group (ventricles: 26 ± 3; atria: 26 ± 3) compared with the control group (ventricles: 8 ± 1, *p* < .0001; atria: 12 ± 1, *p* = .001) (Figure 2F). Liraglutide treatment decreased the number of VCAM‐1+ blood vessels/cm^2^ (ventricles: 16 ± 2; atria: 20 ± 6) compared with the DM rats, but only significant in the ventricles (*p* = .01).

### 3.4 Liraglutide attenuates DM‐induced CML, NOX2 and NOX4 accumulation in the microvasculature of the brain

#### 3.4.1 CML

**FIGURE 3:**
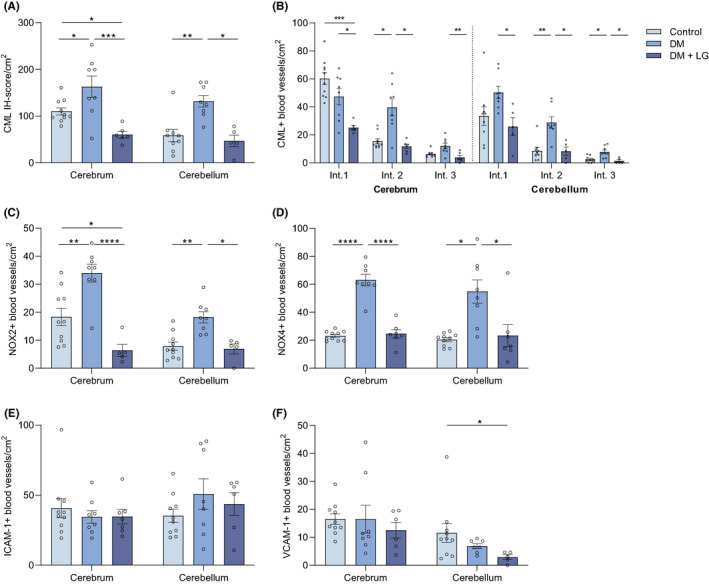
Liraglutide treatment decreases CML, NOX2 and NOX4 accumulation in the microvasculature of the brain in diabetic rats. The immunohistochemical score/cm^2^ for CML ((A) defined in Methods) and the number of positive blood vessels/cm^2^ for NOX2 (C), NOX4 (D), ICAM‐1 (E) and VCAM‐1 (F) of the microvasculature of cerebrum and cerebellum in control rats, STZ‐induced diabetic rats (DM) and Liraglutide‐treated DM rats (DM + LG). The total number of blood vessels with a particular CML intensity score is separately depicted in (B): weak (score 1), moderate (score 2) and strong (score 3) intensity score. A one‐way ANOVA was used for analysis; data are represented as mean ± standard error. *p* < .05*, *p* < .01**, *p* < .001***, *p* < .0001****

Similar to the heart, diabetes induced a significant increase in the presence of CML (IH score/cm^2^) in the microvasculature of the brain (cerebrum: 163 ± 23, *p* = .03; cerebellum: 132 ± 12, *p* = .008), compared with controls (cerebrum: 110 ± 8; cerebellum: 59 ± 13)(Figure 3A). Also here Liraglutide treatment led to a significantly decreased IH score/cm^2^ (cerebrum: 60 ± 7, *p* = .0005; cerebellum: 47 ± 13, *p* = .02) compared with the nontreated DM rats. The CML intensity scores showed that diabetes especially increased the numbers of moderate‐ (intensity score 2) and strong‐positive (intensity score 3) blood vessels in the brain (Figure 3B). Similar to the heart, Liraglutide treatment significantly lowered the number of CML+ blood vessels among all intensity scores in the brain.

#### 3.4.2 NOX2 and NOX4

Diabetes also induced significant increases in the number of NOX2+ (cerebrum: 34 ± 3, *p* = .003; cerebellum: 18 ± 2, *p* = .003) (Figure 3C) and NOX4+ (cerebrum: 63 ± 4, *p* < .0001; cerebellum: 52 ± 9, *p* = .02) (Figure 3D) blood vessels/cm^2^ compared with controls. Liraglutide significantly decreased the number of NOX2+ (cerebrum: 6 ± 2, *p* < .0001; cerebellum: 7 ± 2, *p* = .02) and NOX4+ (cerebrum: 24 ± 3, *p* < .0001; cerebellum: 23 ± 8, *p* = .02) blood vessels/cm^2^ compared with the DM group.

#### 3.4.3 ICAM‐1 and VCAM‐1

In contrast to the heart, diabetes did not affect the numbers of ICAM‐1+ (Figure 3E) or VCAM‐1+ (Figure 3F) blood vessels in the brain. Liraglutide treatment did also not affect the numbers of ICAM‐1+ and VCAM‐1+ blood vessels compared with DM rats, albeit in the cerebellum the number of VCAM‐1+ blood vessels/cm^2^ was significantly lower than in the control group (*p* = .03).

### 3.5 Liraglutide attenuates DM‐induced ICAM‐1 and VCAM‐1 accumulation in the microvasculature of the kidney

#### 3.5.1 ICAM‐1 and VCAM‐1

**FIGURE 4:**
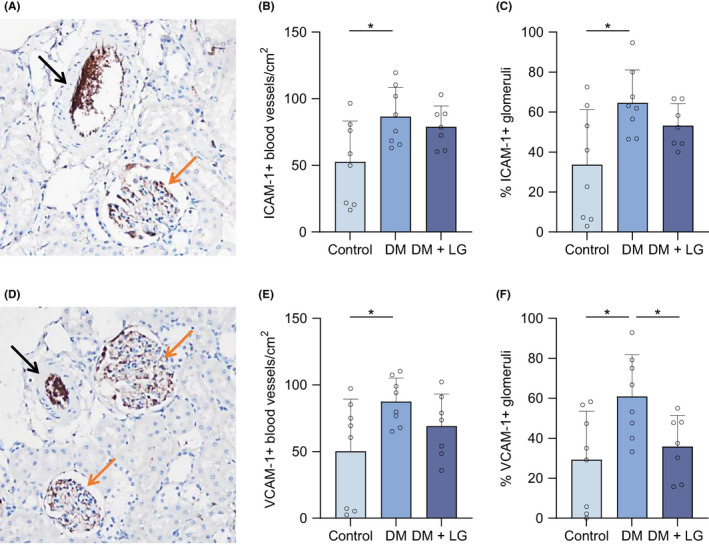
Liraglutide treatment decreases ICAM‐1 and VCAM‐1 accumulation in the microvasculature of the kidney in diabetic rats. Example of a positive ICAM‐1 (A) and VCAM‐1 (D) staining in renal vasculature, i.e., in blood vessels (black arrow) and glomeruli (orange arrow). Original magnification: 200×. The number of positive blood vessels/cm^2^ for ICAM‐1 (B) and VCAM‐1 (E), and the percentage of ICAM‐1‐positive (C) and VCAM‐1‐positive glomeruli (F), was determined in the renal microvasculature in control rats, STZ‐induced diabetic rats (DM) and Liraglutide‐treated DM rats (DM + LG). An unpaired t‐test was used for analysis; data are represented as mean ± standard error. *p* < .05*

In Figure 4, examples are shown of the immunohistochemical stainings of ICAM‐1 (Figure 4A) and VCAM‐1 (Figure 4D) in the endothelium of renal blood vessels and glomeruli.

Diabetes induced a significant increase in the number of ICAM‐1+ blood vessels/cm^2^ (controls: 52.6 ± 30.7; DM group: 86.6 ± 21.8, *p* = .02) (Figure 4C) and the percentage of ICAM‐1+ glomeruli (controls: 33.7 ± 25.6; DM group: 64.6 ± 16.4, *p* = .02) (Figure 4D). Similarly, the number of VCAM‐1+ blood vessels/cm^2^ and the percentage of VCAM‐1+ glomeruli were significantly higher in the diabetic rats (87.6 ± 17.6, *p* = .03; 61 ± 20.8, *p* = .01, respectively) than in the nondiabetic controls (50.3 ± 39.1; 29.3 ± 24.2, respectively) (Figure 4E, F). Liraglutide treatment decreased the number of ICAM‐1+ and VCAM‐1+ blood vessels/cm^2^ and the percentage of ICAM‐1+ and VCAM‐1+ glomeruli, although only the latter achieved statistical significance (35.9 ± 15.6, *p* = .02).

## 4 DISCUSSION

In this study, we found an increased presence of established microvascular dysfunction markers in the heart, brain and kidney of rats with STZ‐induced DM. Liraglutide treatment significantly counteracted these DM‐induced markers both in the heart and brain. Notably, blood glucose levels and bodyweight were not significantly affected by Liraglutide.

It is widely recognised that diabetes is associated with endothelial dysfunction in various vascular beds, including the heart and brain. Our study shows that STZ‐induced loss of glycaemic control resulted in an increased presence of markers associated with glycation (CML), oxidative stress (NOX2 and NOX4 [mainly in the brain]) and inflammation (ICAM‐1 and VCAM‐1 [in the heart only]) in the heart and brain microvasculature. Although we did not determine microvascular function in this study, nor putative consequences on heart or brain function, it is well established that DM‐induced increases in microvascular oxidative stress, glycation and inflammation contribute to an impeded microcirculatory regulation such as vascular dilation and barrier function in the heart^6^ and the brain^2^ in vivo.

Previous studies have shown a causal relation between glycation, oxidative stress and inflammation. Oxidative stress for example was shown to be involved both in the induction and the consequences of AGE formation. CML formation was severely impaired in neutrophils from NOX2‐knockout mice, implying that NOX2‐derived ROS are involved in AGE formation.^18^ Moreover, AGEs were shown to activate both NOX2^19^ and NOX4^20^ in human endothelial cells that, respectively, resulted in increased VCAM‐1 expression and increased endothelial permeability. Whether such causal relations played a role in our model remains to be established. The differences we observed in the presence of these markers between the heart and brain suggest that an interrelatedness in the induction of these markers in endothelial cells may be organ‐dependent.

Liraglutide treatment significantly decreased the DM‐induced microvascular dysfunction markers in our rat model, both in the heart and in the brain, without an apparent effect on blood glucose levels. GLP‐1, and its analogue Liraglutide, is able to lower blood glucose levels by increasing insulin production and release by beta cells and by increasing insulin sensitivity. However, in our study, we used a model of type 1 diabetes, wherein hyperglycaemia is the result of a decreased insulin production after streptozotocin‐induced beta‐cell destruction. This indicates that these effects of Liraglutide were independent of a putative glucose‐lowering effect. In line herewith, in a similar STZ‐induced type 1 diabetes rat model Liraglutide was shown to normalise DM‐induced myocardial NAD(P)H oxidase activity, oxidative stress markers and apoptosis, without affecting plasma glucose and insulin levels.^27^, ^28^ Moreover, multiple studies have shown that Liraglutide can counteract high glucose‐induced oxidative stress, NOX expression, ER stress and adhesion molecule expression in human endothelial cells in vitro,^29^, ^30^ further underscoring that Liraglutide can directly affect endothelial cells. Liraglutide most likely elicits these effects through its agonistic interaction with the GLP‐1 receptor (GLP‐1R). For instance, it was shown in patients with type 2 diabetes that Exenatide, another GLP‐1 analogue, increased endothelial function, as assessed in vivo by peripheral arterial tonometry and ex vivo on human subcutaneous adipose tissue arterioles and endothelial cells.^31^ This effect was abolished by the GLP‐1R antagonist exendin‐9. Similarly, the inhibition of endoplasmatic reticulum stress and the restoration of insulin‐stimulated eNOS activation induced by Liraglutide in isolated endothelial cells from DM patients were abolished by exendin‐9 pretreatment.^32^ However, while GLP‐1R RNA was found in human whole heart and brain tissue,^33^ no GLP‐1 mRNA could be detected in cardiac endothelial cells.^34^ Due to the questionable specificity of the currently available antibodies, whether GLP‐1R is expressed in the vascular endothelium of animals or humans remains to be conclusively determined.^35^ Interestingly, Lixisenatide, another GLP‐1 analogue, reduced apoptosis and increased fractional shortening in cardiomyocytes isolated from wild‐type mice, but also from GLP‐1R knockout mice, indicating the existence of GLP‐1R‐independent mechanisms, which might also apply to the vascular endothelium.^36^

While Liraglutide is registered as a treatment for patients with type 2 diabetes, the application of Liraglutide in patients with type 1 diabetes has been studied as well.^37^, ^38^, ^39^ These studies reveal a decrease in insulin dose requirement and modest improvements in glycaemic control. While we omitted insulin treatment due to our focus on Liraglutide treatment, including insulin treatment in future studies is warranted to address the interplay between Liraglutide, insulin and microvascular dysfunction. Additionally, Liraglutide treatment led to significant weight loss in patients with type 1 diabetes and is in fact now also a registered drug to treat obesity, regardless of DM. In our rats, however, Liraglutide treatment did not result in weight loss. This difference may be due to the fact that most of the studies in patients with type 1 diabetes were performed in overweight/obese patients,^37^, ^38^, ^39^ while our rats were lean. However, the results of Frandsen et al. argue against this explanation as in their study, which specifically excluded obese patients, Liraglutide treatment resulted in a significant weight loss as well.^40^ Furthermore, they found no correlation between baseline BMI and weight loss, indicating that weight loss may be independent of start weight.

A limitation of this study is that we did not measure microvascular function parameters in the animals, such as barrier function and vasomotor function in the heart, brain and kidney. Although AGE accumulation and upregulation of NOX proteins and adhesion molecules have been repeatedly shown to coincide with vascular function impairment ^12^, ^14^, ^17^, ^18^, ^19^, ^20^, ^21^, ^22^, the increased presence of these markers in the microvasculature does not prove dysfunction by itself, nor which functional parameters are impaired and to what degree. Nevertheless, the LEADER trial showed that in addition to a lower risk of cardiovascular morbidity and mortality, Liraglutide treatment reduced microvascular events in the kidneys and decreased the development and progression of diabetic kidney disease in patients with type 2 diabetes.^8^, ^41^

So far, the effects of GLP‐1 analogues on cardiovascular outcomes in patients with type 1 diabetes have not been reported yet. However, type 1 diabetes still poses a greatly increased risk for cardiovascular disease and mortality, particularly in younger patients (<50 years).^42^ Additionally, Rawshani et al. showed recently that this excess risk was related to age at onset in young adults with type 1 diabetes, with groups having up to 18 life‐years lost.^43^ These findings warrant the need for earlier cardioprotection and novel therapies.

Our results point to a protective effect of Liraglutide against microvascular dysfunction in a type 1 diabetes model in vivo, indicating that Liraglutide may improve cardiovascular outcomes in patients with type 1 diabetes as well.

## AUTHOR CONTRIBUTIONS

PK, HN and SS conceived the original idea and supervised the project. UB, RE and AK carried out the experiments and performed the analyses with CS. UB and AK wrote the manuscript. All authors were involved in the critical assessment and interpretation of the data and the final manuscript.

## CONFLICT OF INTEREST

The authors have reported that they have no relationships relevant to the contents of this paper to disclose.
